# VDAC regulates AAC-mediated apoptosis and cytochrome *c* release in yeast

**DOI:** 10.15698/mic2016.10.533

**Published:** 2016-08-25

**Authors:** Dário Trindade, Clara Pereira, Susana R. Chaves, Stéphen Manon, Manuela Côrte-Real, Maria J. Sousa

**Affiliations:** 1 Centro de Biologia Molecular e Ambiental (CBMA), Departamento de Biologia, Universidade do Minho, Campus de Gualtar, 4710-057 Braga, Portugal.; 2 Institut de Biochimie et de Génétique Cellulaires (IBGC), UMR5095 CNRS & Université de Bordeaux, 1 Rue de Camille Saint-Saëns, 33077 Bordeaux, France.; 3 I3S-Instituto de Investigação e Inovação em Saúde, Universidade do Porto, Porto, 4200-135, Portugal.; 4 IBMC-Institute for Molecular and Cell Biology, University of Porto, Porto, 4200-465, Portugal.

**Keywords:** AAC, Por1, mitochondria, cytochrome c, acetic acid, Apoptosis

## Abstract

Mitochondrial outer membrane permeabilization is a key event in apoptosis processes leading to the release of lethal factors. We have previously shown that absence of the ADP/ATP carrier (AAC) proteins (yeast orthologues of mammalian ANT proteins) increased the resistance of yeast cells to acetic acid, preventing MOMP and the release of cytochrome *c* from mitochondria during acetic acid - induced apoptosis. On the other hand, deletion of *POR1* (yeast voltage-dependent anion channel - VDAC) increased the sensitivity of yeast cells to acetic acid. In the present work, we aimed to further characterize the role of yeast VDAC in acetic acid - induced apoptosis and assess if it functionally interacts with AAC proteins. We found that the sensitivity to acetic acid resulting from *POR1* deletion is completely abrogated by the absence of AAC proteins, and propose that Por1p acts as a negative regulator of acetic acid - induced cell death by a mechanism dependent of AAC proteins, by acting on AAC - dependent cytochrome *c *release. Moreover, we show that Por1p has a role in mitochondrial fusion that, contrary to its role in apoptosis, is not affected by the absence of AAC, and demonstrate that mitochondrial network fragmentation is not sufficient to induce release of cytochrome *c* or sensitivity to acetic acid - induced apoptosis. This work enhances our understanding on cytochrome *c* release during cell death, which may be relevant in pathological scenarios where MOMP is compromised.

## INTRODUCTION

Mitochondrial outer membrane permeabilization (MOMP) is a key event in mammalian apoptosis processes leading to the release of lethal factors, like cytochrome *c* (cyt *c*), apoptosis inducing factor (AIF) and Endonuclease G, which may activate downstream apoptotic and non-apoptotic death pathways. In mammalian cells, MOMP has been attributed to different mechanisms, namely: i) opening of the permeability transition pore (PTP), ii) formation of pores/channels in the outer mitochondrial membrane, either by Bcl-2 pro-apoptotic family members or ceramide molecules; and iii) interactions between the different processes and components 1,2,3,4. However, the exact mechanisms of MOMP and its regulation remain to be clarified.

The permeability transition pore (PTP) is a pore formed at contact sites between the inner and outer mitochondrial membranes (IMM and OMM) 5 under conditions of elevated matrix Ca^2+^ concentrations, particularly when accompanied by oxidative stress and depletion of adenine nucleotides and Mg^2+^ 6,7. Long-lasting opening of the PTP results in the collapse of the electrochemical proton gradient, ROS accumulation and the equilibration of ionic gradients and solutes across the IMM, which eventually leads to osmotic swelling of the matrix, cristae remodelling and subsequent rupture of the OMM 8.

The exact molecular composition of the PTP is not completely defined and still remains a matter of debate, although it is generally accepted that PTP opening involves a multicomponent protein complex 9. Several different proteins have been considered as either structural or regulatory components of the pore. Among the first recognized as participating in mitochondrial permeabilization were the voltage-dependent anion channel (VDAC) in the OMM 10,11 and the adenine nucleotide translocator (ANT) in the IMM 12. VDAC, also called mitochondrial porin, functions as a low-specificity molecular sieve and is considered responsible for the permeability of the OMM to several small molecules, therefore regulating the flow of metabolites between the cytoplasm and the mitochondrial inter membrane space (IMS) 13. ANT is normally specific to the transport of adenine nucleotides, but a purified and functional ANT can also unselectively permeabilize lipid vesicles in the presence of Ca^2+^. Furthermore, Ca^2+^-induced mitochondrial permeability transition can be modulated by ligands of ANT 6,14,15. These studies initially suggested that VDAC and ANT were constituents of the PTP, but genetic inactivation studies brought new insights into the molecular composition of the PTP. It is now proposed that PTP can result from the formation of a pore by the mitochondrial phosphate (Pi) carrier 16, from aggregation of misfolded and damaged membrane proteins 17, or be composed by dimers of the F_O_F_1_ ATP synthase 18. Despite the multiple models, it is currently generally accepted that ANT and VDAC are non-essential components of the PTP but play important regulatory functions in the apoptotic process 19. However, whether and how these two regulators functionally interact has not been elucidated yet.

Mitochondria of *Saccharomyes cerevisiae* are very similar to those from mammalian cells. In particular, this organism possesses three isoforms of the ADP/ATP carrier (*AAC1*, *AAC2* and *AAC3*) that are orthologues of mammalian ANTs 20,21,22, as well as a porin (Por1p) that is an orthologue of mammalian VDACs, and a second porin homologue, Por2p, which does not evidence channel properties 23,24. A large-conductance unselective channel, having a size similar to the PTP, has also been detected in yeast mitochondria (YMUC). The ability of yeast mitochondria to undergo Ca^2+^-induced permeability transition suggests that the YMUC and mammalian PTP may be the expression of very similar events, originating the concept of “yeast PTP” 25,26,27. Though the exact composition of the YMUC remains to be elucidated, evidence argues against a contribution of AAC and/or porin 28,29. Nevertheless, absence of Por1p is sufficient to alter the pore’s voltage dependence and desensitizes it to Ca^2+^ regulation 30,31. Furthermore, both AAC and Por1p have been implicated in yeast apoptosis induced by different stimuli. While the absence of AAC proteins increased the resistance of yeast cells to acetic acid and diamide (a thioloxidant compound that induces cyt *c* release from mitochondria and cell death), preventing MOMP and the release of cyt *c* from mitochondria during acetic acid - induced apoptosis 32, deletion of *POR1* increased the sensitivity of yeast cells to acetic acid, hydrogen peroxide and diamide 32. AAC proteins therefore seem to act as pro-death molecules and Por1p as a pro-survival protein. However, whether they share the same pathway in the regulation of yeast apoptosis remains to be clarified.

In this study, we sought to determine whether Por1p functionally interacts with AAC proteins, as well as its contribution to cyt *c* release and yeast apoptosis induced by acetic acid treatment. We found that the sensitivity to acetic acid resulting from *POR1* deletion is completely abrogated by the absence of AAC proteins, and a putative regulatory role of Por1p in cyt *c* release from mitochondria depends on the presence of these IMM carriers. This indicates that Por1p may regulate cell survival by acting as a negative regulator of AAC proteins in the apoptotic cascade.

## RESULTS

### Absence of AAC proteins reverses the sensitivity of the Δ*por1* mutant to acetic acid and diamide

We have previously shown that the increased resistance of Δ*aac1/2/3* cells to acetic acid is accompanied by a delay in the appearance of chromatin condensation, DNA strand breaks and loss of membrane integrity, contrasting with the early development of these events in Δ*por1* cells. Additionally, the delay in the emergence of early and late apoptotic markers was associated with an impairment in MOMP and cyt *c* release from mitochondria to the cytosol of acetic acid-treated yeast cells, which led to the conclusion that AAC proteins are required to promote cyt *c* release to the cytosol, and that Por1p contributes to the resistance of yeast to apoptosis in this particular scenario 32. In contrast, deletion of *POR2* did not affect sensitivity to acetic acid - induced cell death (Fig. S1).

**Figure 1:**
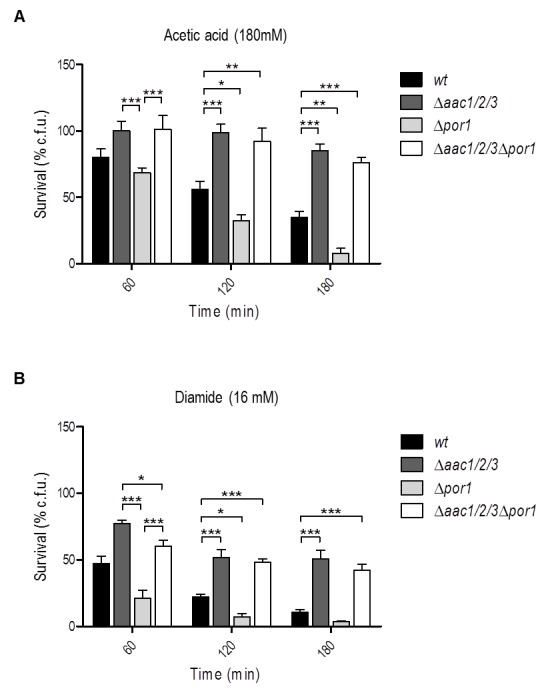
FIGURE 1: Absence of the AAC proteins promotes survival of *S. cerevisiae* to acetic acid and diamide reverting the sensitivity phenotype of Δ*por1* cells. **(A)** Survival of *wt*, Δ*aac1/2/3*, Δ*por1* and Δ*aac1/2/3*Δ*por1* cells after treatment with acetic acid (180 mM) was determined by c.f.u. counts after 60, 120 and 180 minutes of treatment, considering the total c.f.u. number at T0 as 100 % survival. **(B)** Survival of *wt*, Δ*aac1/2/3*, Δ*por1* and Δ*aac1/2/3*Δ*por1* cells after treatment with diamide (16 mM) was determined by c.f.u. counts, as previously mentioned. Cells were pre-cultured in YPD, and then grown O.N. in YPGal or YPD until an O.D._640nm_ of 1.5-2.0 was reached. Data represent mean ± SEM of at least 3 independent experiments. Statistical analysis was performed using a Two-way ANOVA and Bonferroni post-tests (P-values: (*) P < 0.05; (**) P < 0.01; (***) P < 0.001).

To study the interaction between AAC and Por1p in yeast apoptosis, the viability of wild type (*wt)*, Δ*por1*, Δ*aac1/2/3* and Δ*aac1/2/3*Δ*por1* strains during acetic acid treatment (180 mM) was evaluated by c.f.u. counting. Interestingly, the simultaneous absence of AAC and Por1p produces a resistance phenotype similar to that of the Δ*aac1/2/3* mutant, in contrast with the sensitivity phenotype exhibited by Δ*por1 *(Fig. 1A), as we had previously reported 32. Indeed, Δ*aac1/2/3* and Δ*aac1/2/3*Δ*por1* strains revealed the highest plating efficiency after a 180 min exposure to acetic acid, exhibiting survival values (approximately 85% and 75%, respectively) not significantly different from each other. On the other hand, the Δ*por1* strain displayed less than 10% survival after a 180 min treatment against approximately 40% of the *wt* (Fig. 1A), thus exhibiting increased sensitivity to acetic acid (P < 0.01). Furthermore, in the Δ*aac1/2/3*Δ*por1* mutant, the time course of cellular events associated with yeast apoptosis induced by acetic acid such as ROS production, chromatin condensation and loss of plasma membrane integrity observed in the Δ*aac1/2/3*Δ*por1* mutant was identical to that previously observed for the Δ*aac1/2/3 *strain (Fig. S2) 32. A similar result was observed when *wt*, Δ*aac1/2/*3, Δ*por1* and Δ*aac1/2/3*Δ*por1* cells were exposed to diamide (16 mM) (Fig. 1B), a thioloxidant compound that induces cyt *c* release from mitochondria and cell death 32,33. Indeed, the absence of the AAC proteins increased the resistance of yeast cells to a lethal concentration of diamide, and reversed the sensitivity phenotype observed in Δ*por1* cells. Such observations suggest that the sensitivity to acetic acid - induced yeast apoptosis and to diamide - induced cell death resulting from Por1p deficiency, and thus the anti-apoptotic role of Por1p, depends on the presence of AAC proteins. These results indicate there is a conserved regulatory system that depends on the interplay between AAC and Por1 proteins, and is capable of regulating the demise of yeast cells in different scenarios.

### Cytochrome *c* release from mitochondria lacking Por1p is impaired in the absence of AAC

**Figure 2:**
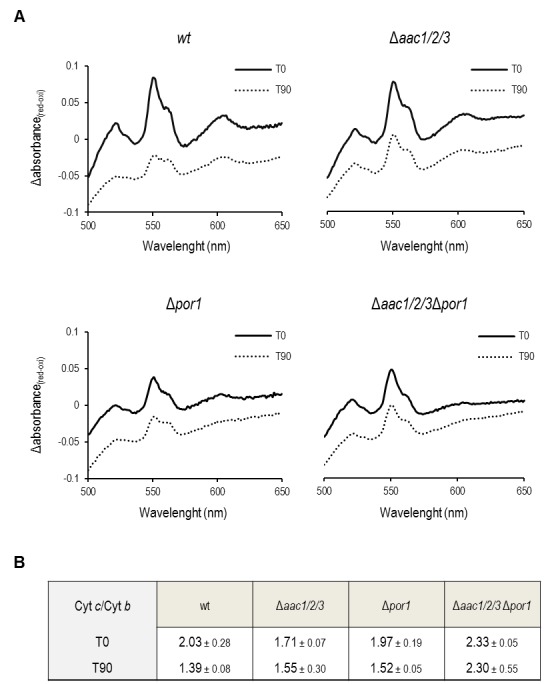
FIGURE 2: Mitochondria lacking the AAC proteins retain most of their cyt *c* after acetic acid treatment. **(A)** Cells were pre-cultured in YPD, and then grown O.N. in YPGal until an OD_640nm_ of 1.5-2.0 was reached, before adding acetic acid. Redox difference spectra of mitochondria isolated from *wt*, Δ*aac1/2/3*, Δ*por1* and Δ*aac1/2/3*Δ*por1* control (full lines) or acetic acid-treated cells (dotted lines) are shown. Each graphic corresponds to one representative experiment from each tested strain. Peaks at 500 nm represent the amount of cytochromes *c* + *c1* while peaks at 561 nm represent the content of cytochrome *b*. **(B)** The corresponding cyt *c*/cyt *b* ratios estimated for *wt*, Δ*aac1/2/3*, Δ*por1* and Δ*aac1/2/3*Δ*por1* mitochondria extracted from control and acetic acid-treated (180 mM, 90 minutes) cells are represented in the lower table. Data represent mean ± SD of at least 3 independent experiments.

Since the sensitivity of Por1p deficient cells to acetic acid was not observed in the absence of AAC proteins, the impact of *POR1* deletion on AAC-mediated cyt *c* release during acetic acid treatment was evaluated by redox spectrometry and Western blot of isolated mitochondria. While mitochondria from Δ*por1* untreated cells produced standard spectra, with an estimated cyt *c*/cyt *b* ratio of 1.97 (Fig. 2C) it was not possible to quantify this ratio in mitochondria prepared from Δ*por1* cells exposed to 180 mM acetic acid for 200 min. Indeed, mitochondria integrity and isolation yield under this condition was significantly lower in comparison to the ones obtained with *wt*, Δ*aac1/2/3* and Δ*aac1/2/3*Δ*por1* strains. To overcome this problem, the mitochondrial content of cytochromes *c*+*c_1_* and *b* was quantified by redox spectrophotometry, in mitochondria isolated from *wt*, Δ*por1* Δ*aac1/2/3*, and Δ*aac1/2/3*Δ*por1* cells before and after 90 minutes of exposure to acetic acid. As expected, mitochondria from *wt* control cells showed cyt *c*/cyt *b* ratios of approximately 2.0 (Fig. 2A), while mitochondria from Δ*aac1/2/3* cells exhibited a slightly lower ratio (approximately 1.70). This observation has been previously reported and might be explained by the lower content of cyt *c* in cells lacking the AAC proteins 32,34. Interestingly, mitochondria from cells lacking both AAC1/2/3 and Por1p exhibited a higher cyt *c*/cyt *b* ratio than *wt* mitochondria (≈ 2.3). This could be in part explained by the lower amount of cyt* b* detected in mitochondria from the Δ*aac1/2/3*Δ*por1* mutant strain (not shown). While treatment with acetic acid led to a significant decrease in the cytochrome ratio of Δ*por1* mitochondria, as it did in *wt* mitochondria, a much smaller variation was observed in *Δaac1/2/3* mitochondria (Fig. 2B). Notably, the cyt *c*/cyt *b* ratio of Δ*aac1/2/3*Δ*por1* isolated mitochondria was not altered (Fig. 2B), indicating that, like in Δ*aac1/2/3 *32*, *cyt *c* release is severely impaired in Δ*aac1/2/3*Δ*por1* cells.

**Figure 3:**
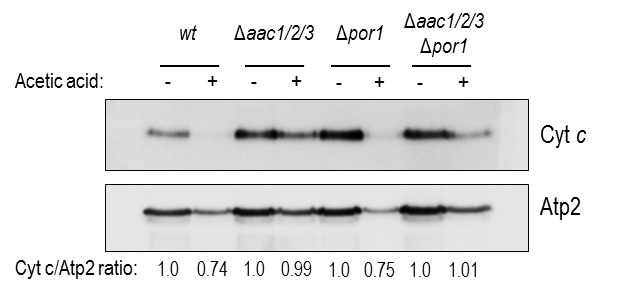
FIGURE 3: Cyt *c* is released from *wt* and Δ*por1* mitochondria. Cells were pre-cultured in YPD, and then grown O.N. in YPGal until an OD_640nm_ of 1.5-2.0 was reached, before adding acetic acid. One representative experiment of cyt *c* immunodetection in *wt*, Δ*aac1/2/3*, Δ*por1*, Δ*aac1/2/3*Δ*por1* mitochondrial fractions, isolated from control and acetic-treated cells, is presented. The beta subunit of the F1 sector of mitochondrial F_O_F_1_ ATP synthase (Atp2p) was used as control for the mitochondrial fractions. A densitometric analysis was performed (ImageJ software) and the corresponding cyt *c* / Atp2 protein ratios are presented.

We confirmed these results by Western blot of mitochondrial samples, where we show a significant decrease of cyt *c* content in mitochondria of acetic acid-treated Δ*por1* cells (Fig. 3), as we previously described for the *wt* strain 32. Indeed, absence of Por1p does not appear to compromise the release of cyt *c*, whose levels are decreased after treatment (approximately 25% less) in both Δ*por1* and *wt* mitochondria (Fig. 3). On the other hand, cyt *c* release from mitochondria of AAC-deficient cells is significantly impaired, and nearly all cyt *c* (approximately 100%) remains in mitochondria after acetic acid treatment (Fig. 3). Likewise, mitochondria from Δ*aac1/2/3*Δ*por1* cells seem to retain all of their cyt *c*, particularly if compared with mitochondria from treated *wt* or Δ*por1* cells (Fig. 3). Taken together, these observations suggest that, following acetic acid treatment, Por1p plays a role in cyt *c* release in an AAC-dependent manner.

### Por1p contributes to the mitochondrial tubular morphology independently of AAC proteins

**Figure 4:**
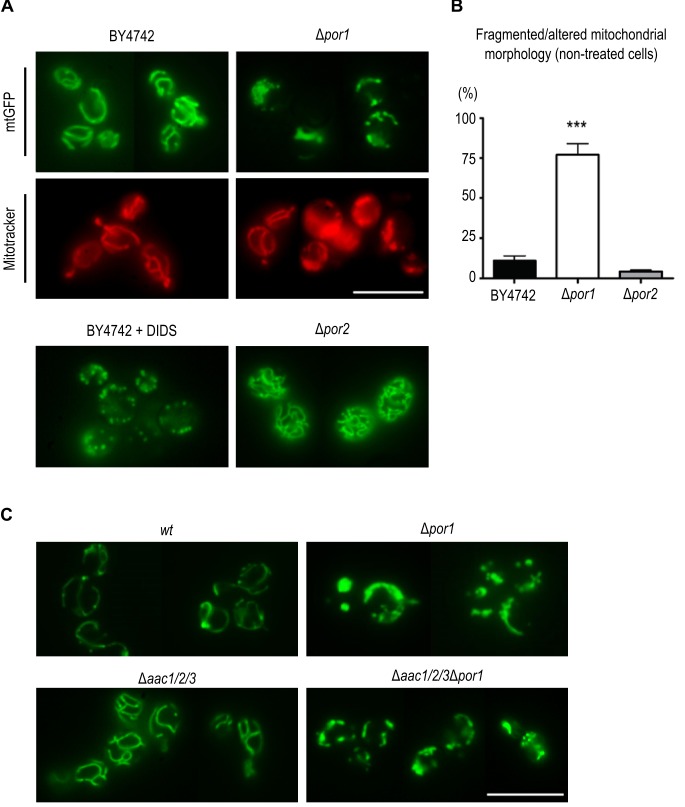
FIGURE 4: Por1p contributes to fusion in non-treated, healthy cells. **(A)** Mitochondrial morphology of BY4742, Δ*por1* and Δ*por2* cells, grown O.N. in SC Gal medium supplemented with the appropriate amino acids, was visualized by expression of a mitochondria matrix-targeted GFP. The BY4742 strain was treated for 5 min with 0.5 mM of the anion channel inhibitor, DIDS. Mitochondrial morphology in BY4742 and Δ*por1* strains was also visualized by Mitotracker Red straining. Bar = 10μm. **(B)** Quantification of fragmented mitochondrial morphology (%) for strains BY4742, Δ*por1* and Δ*por2* is shown. Values are means ± SD of four independent experiments. *** P < 0.001, unpaired t-test. **(C)** Mitochondrial morphology in Δ*aac1/2/3* and Δ*aac1/2/3*Δ*por1* strains expressing a matrix targeted GFP grown O.N. in SC Gal medium supplemented with the appropriate amino acids. Bar = 10 μm.

The collapse of the mitochondrial network into small rounded mitochondria is a common phenomenon in many scenarios of apoptosis (for example 35, 36, reviewed in 37), occurring alongside with MOMP 38. Taking the above observations into account, we sought to assess whether the sensitive phenotype of Δ*por1* cells was related with increased mitochondrial fission, and hence a putative role of Por1p in mitochondrial morphology and its impact on the cellular response to acetic acid. To address this question, we expressed a mitochondria matrix-targeted GFP 39 in *wt* and Δ*por1 *strains, as well as in Δ*por2 *cells, deficient in the second orthologue of VDAC (BY4742, Euroscarf). In the parental strain, exponential phase cells exhibited mitochondria with a normal elongated tubular network morphology (Fig. 4A). In the absence of Por1p, a high percentage of cells presented short spherical mitochondria of different sizes and often aggregated (Fig. 4A and 4C). Treatment of the wild-type cells with DIDS, a compound known to inhibit VDAC 40, led to a quick fragmentation of the mitochondrial network, which supports the view that VDAC contributes to mitochondrial fusion.

To assess whether *POR1* deletion causes complete mitochondrial fragmentation or just fission/constriction of the inner membrane 41,42, mitochondria were stained with Mitotracker Red. This fluorescent probe is mitochondrion-selective and accumulates in response to mitochondrial membrane potential (negative in the matrix side). Mitotracker Red staining of Δ*por1* cells showed a high percentage of cells with fragmented morphology similar to that observed when using the mitochondrial matrix targeted-GFP. This observation indicates that an extensive mitochondrial fragmentation occurs in Δ*por1* mutant mitochondria (Fig. 4A). In contrast, absence of Por2p led to an increase in the extent of the mitochondrial branching in comparison with the parental strain (Fig. 4A).

This effect however is not as dramatic as that observed in mutants impaired in mitochondrial fission, like the Δ*dnm1* strain 43, suggesting that Por2p contributes to this process only to some extent. Quantification of the percentage of BY4742, Δ*por1* and Δ*por2* cells exhibiting fragmented morphology is shown in Fig. 4B. Since the absence of AAC proteins reverted the sensitive phenotype of Δ*por1* mutant to acetic acid - induced apoptosis, we questioned whether it also reverted their fragmented mitochondrial morphology. In contrast with *wt* (W303-1B) and Δ*aac1/2/3* cells, Δ*aac1/2/3*Δ*por1* cells displayed the same fragmented mitochondrial morphology observed in the Δ*por1* mutant, which suggests that the absence of AAC proteins has no effect in mitochondrial network organization (Fig. 4C). These observations suggest that, contrary to its role in apoptosis, the role of Por1p in mitochondrial fusion is not affected by the absence of AAC, and allows dissociating the fragmentation of the mitochondrial network from increased sensitivity to acetic acid and cyt *c* release.

## DISCUSSION 

The mechanisms underlying mitochondrial membrane permeabilization and subsequent release of cyt *c* in response to apoptotic stimuli have been the subject of extensive studies for the last decades. Throughout this period, different components/mechanisms were postulated, including a proposed fundamental role of ANT and VDAC, which affect mitochondrial permeabilization. 44. These proteins were originally thought to compose the scaffold structure of the PTP, but later genetic and molecular studies indicate they are not essential components of the PTP 19,45, and their role in cell death is still poorly understood.

The yeast *S. cerevisiae* has played an important role towards the understanding of mitochondria permeabilization during apoptosis. Indeed, the yeast system has provided relevant insight into mechanisms of mitochondria permeabilization directly mediated by Bax, since it is devoid from obvious orthologues of the Bcl-2 family members, major regulators of mammalian apoptosis and MOMP, with the exception of Ybh3p 46, a non-canonical BH3-only protein 47,48,49 (for a review see 50). Since yeast and mammalian mitochondria are similar and yeast contain orthologues of ANT proteins, porin, F_O_F_1_-ATP synthase and mitochondrial cyclophilin, which are putative components/regulators of the mammalian PTP, here, we further explore the yeast system to understand the involvement of these proteins in MOMP regulation and subsequent release of cyt *c*, independently of Bax and other Bcl-2 family proteins.

Like in the case of multicellular organisms, cyt *c* release from mitochondria is a common event in several scenarios of yeast cell death, including deletion of the histone chaperone *ASF1/CIA1*, pheromone- and amiodarone-induced cell death, *CDC48* mutation, and also H_2_O_2_ or acetic acid treatments 32,51,52,53,54 (for a review see 55). We previously found that the release of cyt *c* during acetic acid - induced death of *S. cerevisiae* depends on the presence of the AAC proteins, an observation that correlates with the increased resistance of the Δ*aac1/2/3* mutant to this stress. We also identified Por1p as a key component of acetic acid - induced apoptosis, though with an opposite effect 32. Here, we assessed the response of *S. cerevisiae* cells lacking the AAC proteins and Por1p to apoptosis-inducing concentrations of acetic acid, as well as their ability to release cyt *c* from mitochondria. We found that yeast cells simultaneously depleted of AAC and Por1p exhibit an acetic acid-resistance phenotype, and cell survival identical to the Δ*aac1/2/3 *strain. This effect is not exclusive to acetic acid, since the absence of AAC proteins can also protect yeast cells from death induced by diamide, a thioloxidant compound, as well as prevent the sensitivity phenotype resulting from the absence of Por1p. We also show that deletion of Por1p does not affect cyt *c* release in the Δ*aac1/2/3* background, since mitochondria from Δ*aac1/2/3*Δ*por1* cells, much like those from Δ*aac1/2/3 *cells*,* retain most cyt *c* after exposure to acetic acid. These observations corroborate the hypothesis that the AAC proteins are required for efficient cyt *c* release, unlike Por1p which may play a distinct regulatory function, a scenario that has also been hypothesized in mammalian cells 9. While AAC proteins have a significant impact on yeast cell survival in response to acetic acid, the role of Por1p seems to depend on the presence of the AACs. The fact that the resistance phenotype provided by the absence of AAC proteins actually overcomes the sensitivity phenotype resulting from *POR1* deletion suggests that a regulatory role of Por1p would only be required when the AAC proteins are present in the IMM.

One possibility is that Por1p negatively regulates AAC proteins, either directly or by causing structural changes in mitochondria, counteracting AAC-mediated cyt *c *release. Indeed, absence of Por1p results in increased difficulty to purify intact mitochondria only when AAC proteins are present, suggesting that Por1p can act on the AAC proteins to regulate mitochondria permeability. Although we did not observe differences in the extension of release of cyt *c* in cells lacking Por1p, in comparison to the *wt* strain, the acetic acid sensitive phenotype of these cells and the lability of their mitochondria after acid treatment suggest that cyt *c* release occurs earlier. Nevertheless, we cannot exclude the possibility that Por1p acts downstream of AAC but independently of it.

Por1p is crucial to normal mitochondrial physiology associated with functions as diverse as the maintenance of redox state, mitochondrial DNA import and even cytoskeleton rearrangements 56,57,58. Additionally, we present evidence supporting a role for Por1p in the organization of the mitochondrial network. This contribution of Por1p to mitochondrial morphology might be of outmost importance to understand the role of this protein in yeast apoptosis. In healthy growing cells, absence of Por1p leads to a phenotype of fragmentation of the mitochondrial network, while absence of Por2p, a second VDAC isoform in yeast, leads to a slight increase in the extent of branching. Overexpression of *POR2*, a homolog of *POR1*, corrects the inability of Δ*por1* cells to grow on yeast media containing a non-fermentable carbon source at an elevated temperature (37°C) 24. However, no Por2p channels were detected electrophysiologically in reconstituted systems and its overexpression does not confer additional permeability to liposomes or intact mitochondria 24,59. This evidence indicates that Por2p, unlike Por1p, is not a real porin and does not normally form channels. The different contributions of these proteins to mitochondria morphology further strengthen the idea that the two yeast VDAC isoforms have different, specialized functions.

Remodelling of the mitochondrial membrane has been suggested to play a role in the release of cyt *c* in mammalian cells 60,61. Accordingly, changes in mitochondria morphology dynamics might also modulate apoptosis in yeasts. Deletion of yeast fission proteins Dnm1p or Mvd1p delays the fragmentation of the mitochondrial network and, in case of cells lacking Mvd1p, promotes cell survival following a death stimulus or in ageing cells 62,63. Ysp1p 53, Ysp2p 64 and Yca1p 65 are all required for fission of the mitochondrial network during apoptosis and their absence leads to apoptosis resistance. Mammalian proteins associated with the release of cyt* c* were also shown to affect mitochondria morphology 66,67, creating a possible link between apoptosis, cyt *c* release and fragmentation of the mitochondrial network. We therefore hypothesised that the highly fragmented mitochondrial network in the Δ*por1* strain could contribute to the increase susceptibility of this strain to apoptosis, for example by facilitating cyt *c* release. Indeed, our data suggest that after 90 minutes of exposure to acetic acid, mitochondria from *wt* and Δ*por1* cells display a reduction in the level of cytochrome *c*. However, the fact that the Δ*aac1/2/3*Δ*por1* mutant still displays a highly fragmented mitochondrial network but has an impaired release of cyt *c* allows ruling out the fragmented phenotype as the cause of cyt *c* release.

In mammalian cells, VDAC was implicated in the association of mitochondria with the cytoskeleton 68. Disruption of the interaction of mitochondria with the cytoskeleton alters the normal mitochondrial morphology, giving origin to a fragmented mitochondrial network 69. However, Blachly-Dyson and colleagues 24 reported that Por1p is not necessary for yeast mitochondrial segregation into the daughter cell, casting doubts on a role of Por1p in actin binding. As such, it will be important to assess if the destabilization of the mitochondrial network in the absence of Por1p is due to improper binding of mitochondria to the actin cytoskeleton or if Por1p is interfering with the organelle fission/fusion machinery.

In summary, we propose that Por1p acts as a negative regulator of cyt *c* release from mitochondria of yeast cells exposed to acetic acid by counteracting AAC - dependent cyt *c *release. Moreover, we show that Por1p has a role in mitochondrial morphology that, contrary to its role in apoptosis, is not affected by the absence of AAC, and demonstrate that mitochondrial network fragmentation is not sufficient to induce release of cyt *c* or sensitivity to acetic acid - induced apoptosis.

This work enhances our understanding on cyt *c* release, which may be relevant for mammalian cells, namely in pathological scenarios where MOMP is compromised, such as cancer 70.

## MATERIALS AND METHODS

### Strains and Growth Conditions

The yeast strains used in this study are listed in Table 1. *Saccharomyces cerevisiae* strains W303-1B and JL1-3*Δ2Δ3*, which is a derivate of W303 lacking the three isoforms of the AAC (*AAC1*, *2* and *3*; 71), were transformed with a Δ*por1::kanMX4* interruption cassette, amplified by PCR from genomic DNA of BY4741 Δ*por1* EUROSCARF deletion strain (EUROSCARF, Institute of Molecular Biosciences Johann Wolfgang Goethe-University Frankfurt, Germany), to generate Δ*por1* and Δ*aac1/2/3*Δ*por1* strains, respectively. Yeast cells were transformed by the lithium acetate method 72, selected in medium containing geneticin (200 µg/µL) and confirmed by PCR. For all experiments performed with these strains, cells were pre-grown in YPD medium (2% Glucose, 1% yeast extract, 1% bactopeptone), transferred to YPGal medium (2% Galactose, 1% yeast extract, 1% bactopeptone) and incubated over-night (O.N.) at 30°C (200 r.p.m.) until an optical density of 1.5-2.0 was reached, essentially as previously described 32. Strains BY4742 *wt*, BY4742 Δ*por1* and BY4742 Δ*por2* were obtained from the EUROSCARF’s gene deletion library and transformed, along with Δ*aac1/2/3* and Δ*aac1/2/3*Δ*por1*, with plasmid YX232-mtGFP, containing the sequence of a mitochondria-targeted GFP 39. These strains were grown in synthetic complete (SC) medium (0.67% Bacto yeast nitrogen base w/o amino acids, 2% (w/v) Galactose, 0.2% (w/v) Dropout mix) supplemented with the appropriate amino acids.

**Table 1:** Yeast strains.

**Name**	**Genotype**	**Source/Reference**
*wt*	W303-1B (*MATα; ura3; trp1; leu2; his3; ade2; canR*)	Gift from Alexander Tzagoloff
Δ*aac1/2/3*	JL1-3Δ2Δ3 (*MATα, ade2, his3; leu2; trp1; ura3; can1; *Δ*aac1::LEU2; *Δ*aac2::HIS3; *Δ*aac3::URA3*)	Postis *et al.*, 2005
Δ*por1*	W303-1B; Δ*por1::kanMX4*	This study
Δ*aac1/2/3*Δ*por1*	JL1-3Δ2Δ3; Δ*por1::kanMX4*	This study
BY4742 *wt*	BY4742 (*MATα ; his3; leu2; lys2; ura3*)	Euroscarf
BY4742 Δ*por1*	BY4742 (*MATα ; his3; leu2; lys2; ura3; YNL055c::kanMX4/YNL055c*)	Euroscarf
BY4742 Δ*por2*	BY4742 (*MATα ; his3; leu2; lys2; ura3; YIL114c::kanMX4*)	Euroscarf

### Acetic acid and Diamide treatments 

For acetic acid tolerance and mitochondria extraction assays, acetic acid was added at a final concentration of 180 mM to cultures grown O.N. until exponential growth phase (OD_640nm_ = 1.5-2.0) in YPGal medium. For diamide survival assays, a working solution of diazenedicarboxylic acid bis 5 N,Ndimethylamide (diamide, from Sigma) was prepared in water (1 M), and added to cultures grown O.N. until exponential growth phase (OD_640nm_ = 1.5-2.0) in YPD medium, to a final concentration of 16 mM. Viability of *wt*, Δ*aac1/2/3*, Δ*por1* and Δ*aac1/2/3*Δ*por1* cells during acetic acid and diamide treatments was evaluated by colony forming units (c.f.u.) counting. Samples collected at different time points during a 3 hours period were diluted, plated onto YPDA (YPD supplemented with 2% Agar; 200 μL from a 1.25x10^3^ cell/mL suspension) and grown for 2 days at 30°C. Percentage of viable cells was estimated considering 100% survival the number of c.f.u. at time zero minutes, right before the addition of acetic acid.

### Mitochondria Isolation

To isolate yeast mitochondria, approximately 5 L of *wt*, Δ*aac1/2/3*, Δ*por1* and Δ*aac1/2/3*Δ*por1* cultures were grown O.N. in YPGal as previously described. Half of each culture was harvested and used as control (T0), while the remaining was subjected to acetic acid treatment (180 mM) for 90 minutes. Control and acetic acid treated cells were converted into spheroplasts by enzymatic digestion with zymolyase (Zymolyase 20T, Seikagaku Biobusiness Corporation), disrupted by hand-potter or mechanical homogenization, and the mitochondrial fraction recovered after a series of differential centrifugations 73. Mitochondrial suspensions were frozen in liquid nitrogen and stored at -80°C. All protein quantifications were performed by the Lowry method 74.

### Redox Spectrophotometry

A mitochondrial suspension with the final concentration of 10 mg/mL of mitochondria protein in recuperation buffer (0.6 M Mannitol; 10 mM Tris-maleate; 2 mM EGTA; pH 6.8) was prepared and equally divided into two eppendorf tubes. The reference and sample tubes were oxidized and reduced with potassium ferricyanide and sodium dithionite, respectively. Sample absorbance was measured using a micro-plate spectrophotometer, and the redox difference spectra were acquired between 500 and 650 nm. Cytochromes *c*+*c_1_*, *b* and *a*+*a_3_* were quantified by the OD differences, 550 nm minus 540 nm, 561 nm minus 575 nm, and 603 nm minus 630 nm, respectively.

### Western blot analysis

For characterization of the mitochondrial fractions by Western blot, 50 μg of proteins were precipitated with TCA, and solubilized in 2% SDS before being separated by SDS-PAGE 75. Proteins were then blotted onto PVDF membranes. Characterization was carried out with antibodies directed against cytochrome *c* (rabbit polyclonal, 1:1000, custom-made by Millegen) and against the beta subunit of the F1 sector of mitochondrial F_O_F_1_ ATP synthase (rabbit polyclonal, 1:20000, home made by Jean Velours, IBGC, Bordeaux).

### Fluorescence microscopy

Cells transformed with the plasmid YX232-mtGFP were grown O.N. as previously described, collected and immobilized in the slides by adding 0.5% (w/v) agar prior to microscopy. When used, MitoTracker Red CMXRos (Molecular Probes) was added to the culture medium at a final concentration of 0.4 µg/mL and incubated for 20 min at 37° C. For the assays with the anion channel inhibitor 4´-diisothiocyano-2,2´-disulfonic acid stilbene (DIDS), overnight grown cells were harvested and incubated in growth medium with 0.5 mM of DIDS for 5 min 40. Samples were analysed on a Leica Microsystems DM-5000B epifluorescence microscope with appropriate filter settings using a 100 x oil-immersion objective. Images were acquired with a Leica DCF350FX digital camera and processed with LAS AF Leica Microsystems software (Leica Mycrosystems).

## SUPPLEMENTAL MATERIAL

Click here for supplemental data file.

All supplemental data for this article are also available online at http://microbialcell.com/researcharticles/vdac-regulates-aac-mediated-apoptosis-and-cytochrome-c-release-in-yeast/.
