# Supplementary material for: VDAC regulates AAC-mediated apoptosis and cytochrome c release in yeast
Source: Microb Cell. 2016 Aug 25;3(10):500–10. doi: 10.15698/mic2016.10.533 (PMC5348984; doi:10.15698/mic2016.10.533)
Supplement: Supplementary file 1 [file mic-03-500-s01.pdf]

Supplemental Material – Figure S1

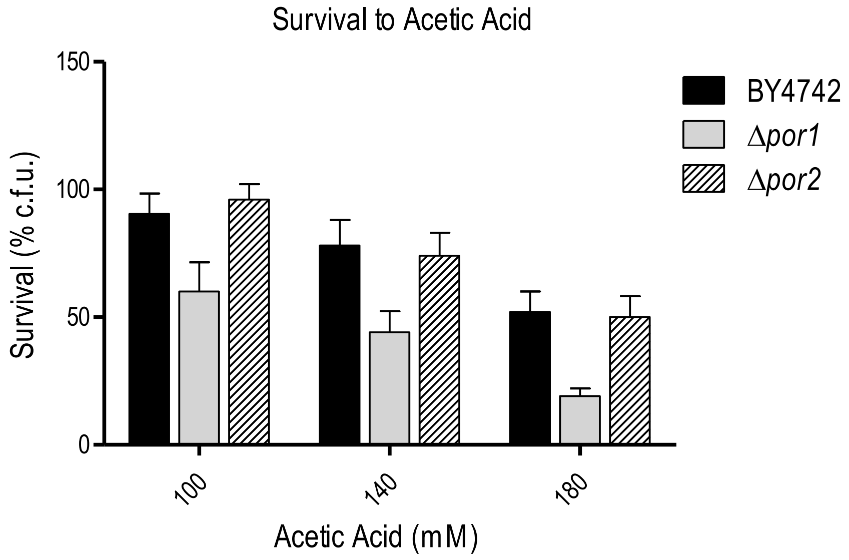

Fig. S1 – Δ*por1*, but not Δ*por2*, mutant exhibits increased cell death in response to different concentrations of acetic acid. Bars represent cell survival of a wild-type strain (BY4742), Δ*por1* and Δ*por2* after 200 min treatment with 100, 140 and 180 mM acetic acid. Total c.f.u. number at T0 was considered as 100 % survival. Cells lacking Por1p exhibit increased sensitivity to the different acetic acid treatments, when compared to the wild-type strain. On the other hand, absence of Por2p does not affect the resistance of yeast cells to acetic acid, with the survival of Δ*por2* cells being significantly different (higher) from that of Δ*por1* cells, and identical to that of the parental strain. Values are means ± SD of four independent experiments. Statistical analysis was performed using a Two-way ANOVA and Bonferroni post-tests (P-values: (***) $P < 0.001$).

Supplemental Material – Figure S2

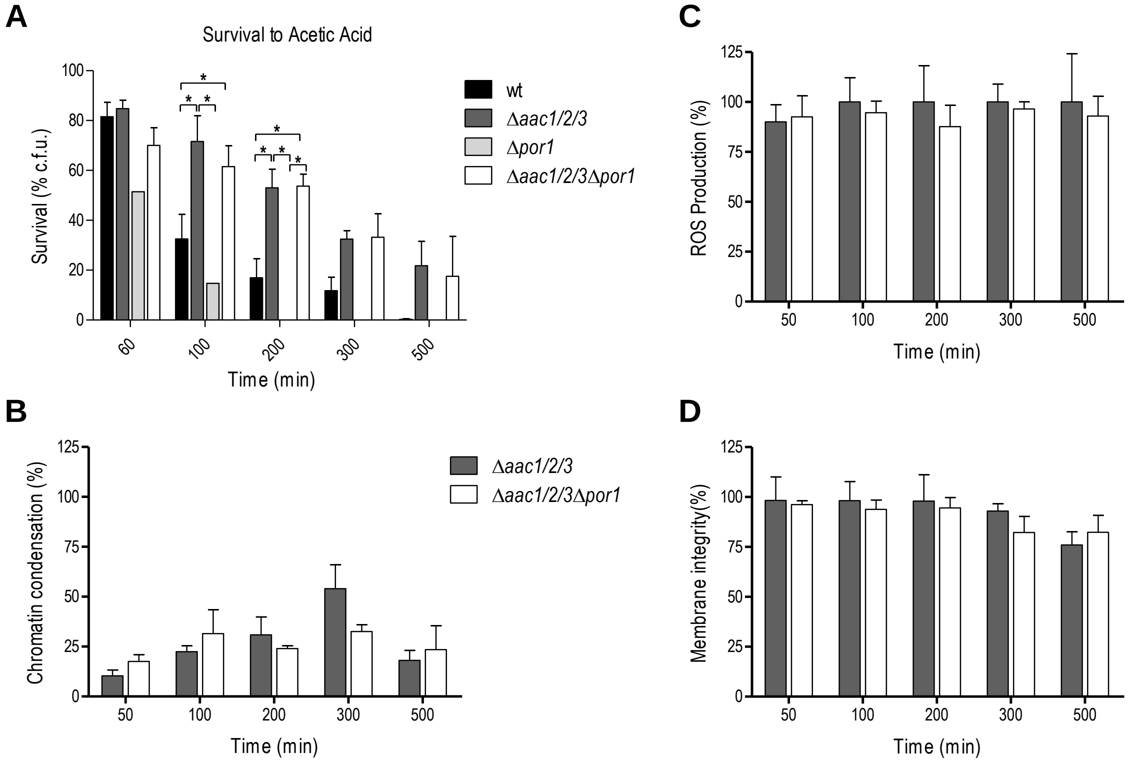

Fig. S2 – Survival of wt, Δ*aac1/2/3*, Δ*por1* and Δ*aac1/2/3*Δ*por1* cells (A), and the kinetic analyses of ROS production (B), chromatin condensation (C) and membrane integrity (D) of Δ*aac1/2/3*Δ*por1* cells was carried out over 500 min acetic acid treatment. Absence of AAC proteins reverts the sensitivity phenotype resulting from *POR1* deletion (P-values: (*) $P < 0.05$). Mitochondrial ROS production, evidenced by MitoTracker Red CM-H2XRos staining, showed a high level of positive cells from the beginning of the treatment, while chromatin condensation using DAPI staining, exhibited a maximum percentage of 40% at 100 min. Finally, loss of membrane integrity using PI staining showed a low level of positive cells indicating that death was not accompanied by loss of membrane integrity. The results showed that as for Δ*aac1/2/3* strain, the higher resistance of Δ*por1*Δ*aac1/2/3* strain is associated with a delayed emergence of apoptotic markers.
